# Regulation and Potential Biological Role of Fibroblast Growth Factor 21 in Chronic Kidney Disease

**DOI:** 10.3389/fphys.2021.764503

**Published:** 2021-10-05

**Authors:** Xue Zhou, Yuefeng Zhang, Ning Wang

**Affiliations:** ^1^Department of Nephrology, Tianjin Haihe Hospital, Tianjin, China; ^2^Tianjin Third Central Hospital, Tianjin, China

**Keywords:** fibroblast growth factor 21, biomarker, chronic kidney disease, diabetic nephropathy, cardiovascular disease

## Abstract

Chronic kidney disease (CKD) is an incurable progressive disease with the progressive impairment of kidney function, which can accelerate the progression of cardiovascular disease, increase the risk of infection, and lead to related complications such as anemia and bone disease. CKD is to a great extent preventable and treatable, and it is particularly important to improve the early diagnosis, strengthen the research underlying the mechanism of disease occurrence and development, and innovate new intervention measures. Fibroblast growth factor 21 (FGF21) belongs to one of members of endocrine FGF subfamily with evolutionarily conserved functions and performs a vital role in the regulation of energy balance and adipose metabolism. FGF21 needs to rely on β-Klotho protein to specifically bind to FGF receptor (FGFR), which activates the FGF21 signaling exerting the biological function. FGF21 is deemed as an important regulatory factor extensively modulating many cellular functions under physiologic and pathologic conditions. Although the metabolic effect of FGF21 has been extensively studied, its potential biological role in the kidney has not been generally investigated. In this review, we summarize the biological characteristics, regulation and biological function of FGF21 based on the current studies, and briefly discuss the potential relationship with chronic kidney disease.

## Introduction

Chronic kidney disease (CKD) is a progressive disease characterized by high morbidity and mortality, which is characterized by the changes in the structure and function of the kidney owing to various reasons (Kalantar-Zadeh et al., 2021). With the high and still increasing global burden of CKD, approximately 10% of adults are affected by CKD (GBD Chronic Kidney Disease Collaboration, 2020). Overall speaking, the incidence of CKD increases with age, especially in patients with obesity, diabetes and hypertension (Silverwood et al., 2013; Cockwell and Fisher, 2020). CKD can accelerate the progression of cardiovascular disease, increase the risk of infection, and lead to anemia and bone disease, as well as other complications that increase the risk of premature death. Since the progression of CKD usually takes several years and the symptoms remain not obvious, early detection of the disease is particularly important. Non-drug strategies such as diet and lifestyle adjustments, and specific drug interventions can be adopted to improve the clinical outcomes of the patients with CKD.

In the past few decades, some new biomarkers have been discovered, which contributes to recognizing renal function impairment earlier. Fibroblast growth factor 21 (FGF21), one of the emerging biomarkers, has been associated with CKD (Kondo et al., 2020). Studies have verified that serum FGF21 levels in patients with CKD increase progressively and reach 20 times over the normal range (Hindricks et al., 2014). Moreover, in type 2 diabetes patients, the level of serum FGF21 is significantly linked to the occurrence of nephropathy, proteinuria, and the progression of end-stage renal disease (ESRD) (Jian et al., 2012; Lee et al., 2015). This article will review the biological characteristics, regulation and biological function of FGF21 in the onset and development of CKD, and finally evaluate the underlying role as a therapeutic target.

## Biological Characteristics of Fibroblast Growth Factor 21

### Molecular Structure of Fibroblast Growth Factor 21

As a member of the superfamily of fibroblast growth factors, FGF21 is composed of similar structure containing 150–300 amino acids (Itoh and Ornitz, 2011). FGF21, initially discovered in 2000, was most similar to FGF19 with approximately 35% similarity in the members of human FGFs (Nishimura et al., 2000). FGF21 precursor is composed of 209 amino acids encoded by 4 exons. Next, undergoing cleavage of a signal peptide, FGF21 precursor is converted into mature FGF21 containing 181 amino acids with molecular weight of approximately 20 kDa (Zhang et al., 2015).

### Fibroblast Growth Factor 21 Related Receptors

Studies have found that FGF exerts biological functions by binding to FGF receptors (FGFRs) belonging to tyrosine kinase receptors. However, the endocrine FGF (FGF19, FGF21, and FGF23) has a low affinity with FGFRs, and requires the participation of specific transmembrane glycoproteins (α or β-klotho) in the target organs. Klotho protein is an important part of the endocrine FGF receptor complex and is indispensable to the high-affinity binding between FGF and FGFR (Fernandes-Freitas and Owen, 2015; Kuro-O, 2019b). In recent years, a growing body of researches have revealed that the FGF-Klotho complex also participates in the pathophysiology of some diseases, involving CKD, diabetes, arteriosclerosis and cancer (Kuro-o, 2012). Consequently, through the thorough research on the FGF-Klotho-FGFR complex, the development of drugs targeting the FGF-Klotho endocrine axis may bring clinical benefits in multiple systems.

The specific binding between FGF21 and corresponding FGFR relies on β-Klotho protein (Ding et al., 2012). β-Klotho protein preferentially combines with FGFR for inhibiting paracrine FGFs signaling (Goetz et al., 2007), which helps endocrine FGFs to specifically bind to FGFRs in target cells avoiding the interference of paracrine FGFs. FGF signaling pathway with abundant structural information extensively modulates distinct biological process in development, tissue homeostasis and metabolism (Goetz and Mohammadi, 2013). In liver, FGF21 participates in regulating carbohydrate and fatty acid metabolism, including fatty acid oxidation and gluconeogenesis (Potthoff et al., 2009). In addition, FGF21 increases glucose uptake and lipolysis through converting white fat into brown fat for increasing energy metabolism (Emanuelli et al., 2014).

### Regulation and Function of Fibroblast Growth Factor 21

**FIGURE 1:**
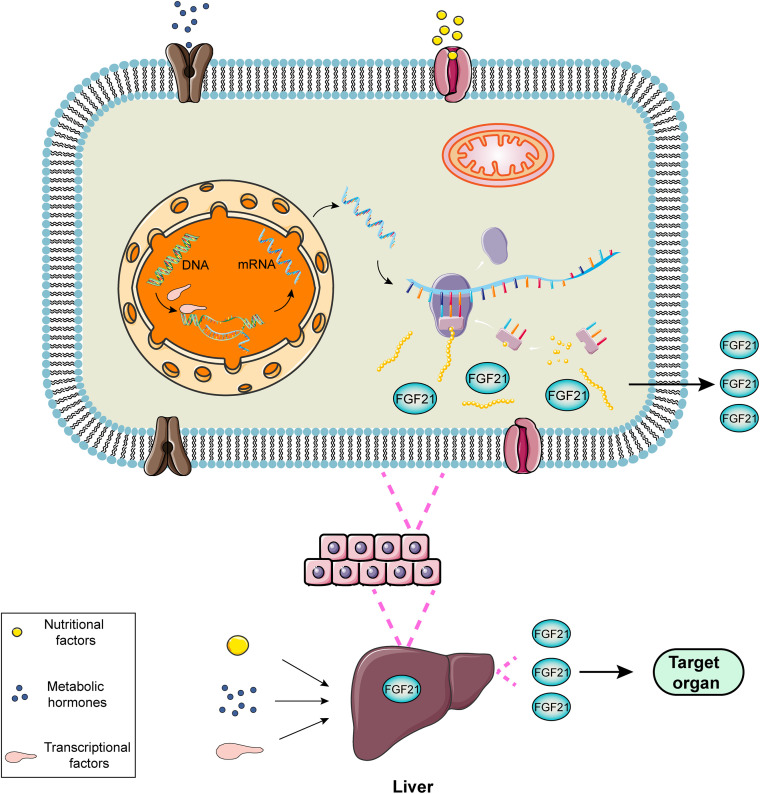
Schematic diagram of the regulatory process of FGF21 expression. FGF21 is a liver-derived hormone regulated by some elements such as nutritional factors, metabolic hormones or transcriptional factors. These factors contribute to improving the transcription and translation process and promoting FGF21 production via activating corresponding signaling pathways. FGF21 secreted from the liver performs potential biological roles in the target organs.

FGF21, a hormone mainly produced in liver, is induced directly by peroxisome proliferator-activated receptor-α (PPARα) (Inagaki et al., 2007). In the fasting or starvation state, PPAR mediates the increase in the expression of FGF21 in the liver, leading to gluconeogenesis, fatty acid oxidation and ketone body production as an adaptive response to hunger (Woo et al., 2013). PPARα agonists, such as bezafibrate and a novel drug MHY2013, can significantly increase the expression of FGF21 for alleviating obesity-induced insulin resistance, dyslipidemia and hepatic steatosis (An et al., 2017). The regulatory process of FGF21 expression can be exhibited utilizing a diagram (Figure 1).

Recently, the biological characteristics of FGF have been extensively studied. The endocrine FGFs play multifaceted roles in the treatment of many chronic diseases involving in kidney disease, cardiovascular disease, obesity, type 2 diabetes, and cancer (Degirolamo et al., 2016). The abnormal signal of FGF is significantly linked to the development of cancer and metabolic diseases. The function of FGF21 was originally discovered in 2005 when looking for new drugs for the treatment of diabetes. FGF21 metabolic axes extensively participates in regulating the metabolic homeostasis (Li, 2019). FGF21 can improve glucose uptake by fibroblasts and adipocytes showing the characteristics of effective treatment of diabetes (Kharitonenkov et al., 2005). At present, FGF21, deemed as a metabolism-related hormone, is an emerging therapeutic target for metabolic diseases (Angelin et al., 2012; Lancha et al., 2012; Reitman, 2013). Furthermore, FGF21 can improve tissue damage caused by the harmful effects of metabolic abnormalities, including oxidative, inflammatory, and immune stress state (Luo et al., 2017). Consequently, some targeting FGF21 analogs have been developed for the treatment of metabolic disorders (Zhang and Li, 2015).

The latest research evidence confirms that FGF21 can improve metabolic status with anti-fibrotic effects and has the potential treatment for non-alcoholic steatohepatitis (NASH) (Harrison et al., 2021). A new type of long-acting FGF21 (LAPS-FGF21) has been developed for potential therapeutic effects on obesity. LAPS-FGF21 is chemically coupled with human IgG4 Fc fragment for a longer half-life in the serum, which can effectively reduce body weight and improve glucose tolerance in a dose-dependent manner at the same time (Kim et al., 2021). FGF21 is highly expressed in the exocrine glands of the pancreas, the mechanism of which requires the FGFR-Klotho signaling transduction (Coate et al., 2017). Under physiological conditions, acute exercise can upregulate the expression level of FGF21 in skeletal muscle (Di Raimondo et al., 2016; Tanimura et al., 2016). At the same time, FGF21 has a higher level in patients with mitochondrial diseases affecting skeletal muscle, which can be used as a biomarker for mitochondrial respiratory chain defects in muscles (Suomalainen et al., 2011; Suomalainen, 2013; Nohara et al., 2019).

FGF21 is released by cardiomyocytes for avoiding hypertrophy, and also participates in regulating the expression of antioxidant pathway genes for reducing reactive oxygen species (ROS) mediated oxidative stress in cardiomyocytes, and acting as an antioxidant factor in the heart to control inflammation and cardiac hypertrophy (Planavila et al., 2013, 2015). Moreover, FGF21 can prevent atherosclerosis by regulating the interconnection among adipose tissue, liver and blood vessels (Lin et al., 2015), and activating the angiotensin converting enzyme 2-angiotensin axis for preventing angiotensin II-induced hypertension and vascular damage (Pan et al., 2018). In addition, FGF21 can also play a therapeutic effect on atherosclerosis through the NF-κB pathway (Zhang et al., 2018). During cardiac remodeling in uremic cardiomyopathy, the effect of increased FGF21 expression on cardioprotective is needed to be further clarified (Suassuna et al., 2020). FGF21 partially ameliorates hyperglycemia by reducing renal glucose reabsorption based on the sodium glucose cotransporter 2 (SGLT2) pathway (Li et al., 2018b).

Since FGF21 is mainly excreted by the kidney (He et al., 2018), it can be predicted by the relative change of creatinine. The estimated glomerular filtration rate (eGFR) is a strong independent negative predictor of FGF21. Synergistic therapy of glucagon-like peptide-1 (GLP-1) and glucagon receptors can upregulate the expression of FGF21 and abate renal insufficiency induced by diabetes (Patel et al., 2018). Furthermore, one study has found that FGF21 has a protective effect on kidney against low protein diet-induced renal damage and inflammation (Fang et al., 2021). It has been reported that the level of circulating FGF21 is independently correlated with the occurrence of contrast-induced nephropathy (CIN) and corresponding kidney injury in patients receiving coronary angiography (CAG) (Wu et al., 2018).

## Fibroblast Growth Factor 21 and Diabetic Nephropathy

FGF21 is closely related to metabolic disorders including diabetes. In order to clarify the relationship between FGF21 and blood glucose, the results of a cohort study demonstrated that the level of FGF21 in the plasma of diabetic patients significantly increased, and was identified as an independent predictor of type 2 diabetes predicting the development of diabetes (Chen et al., 2011). A study has found that the high serum FGF21 level is correlated with low urinary glucose excretion (UGE) in type 2 diabetes patients (Zhang et al., 2021). Moreover, a meta-analysis found that FGF21 level in the plasma of type 2 diabetes patients significantly increased compared with the control group, which was affected by the variables of body mass index (BMI), total cholesterol and triglycerides (Wang et al., 2019). There is evidence existing to support that genetic variation in the FGF21 gene region is related to the renal function of type 2 diabetes patients and affects the eGFR of diabetic patients (Yu et al., 2019).

Studies have found that FGF21 levels can be used as a biomarker related to the prognosis of patients with diabetic nephropathy (El-Saeed and El-Mohasseb, 2017; Chang et al., 2021). Serum FGF21 levels are closely associated with early diabetic nephropathy in high-risk groups of type 2 diabetes patients, especially the circulating FGF21 value increasing more than 181 pg/mL, so that effectively targeting FGF21 therapy may contribute to early detection and prevention of diabetic microvessels complication (Esteghamati et al., 2017). A cross-sectional study also confirmed that elevated serum FGF21 level may be a useful biomarker for predicting the progression of kidney disease, especially in the early stage of diabetic nephropathy. Additionally, a recombinant human FGF21, PEGylated rhFGF21 (PEG-rhFGF21), has been developed for the treatment effect on diabetic nephropathy in diet induced obesity animal model (Zhao et al., 2017).

Insulin resistance is a pivotal process in the occurrence and development of diabetic nephropathy. Studies have found that alprostadil (prostaglandin E1) can reduce the insulin resistance via the autophagy-dependent FGF21 pathway for preventing the progression of diabetic nephropathy (Wei et al., 2018). FGF21 can negatively regulate TGF-β-p53-Smad2/3-mediated epithelial-to-mesenchymal transition by activating AKT for reducing diabetes-induced renal fibrosis (Lin et al., 2020). Based on the db/db mouse model research, targeting FGF21 treatment could function as a potential therapeutic strategy in type 2 diabetic nephropathy for significantly down-regulating FGF21 receptor components, activating ERK phosphorylation, reducing the excretion of urinary albumin and mesangial expansion, inhibiting the synthesis of pro-fibrotic molecules, and improving renal lipid metabolism and oxidative stress damage (Kim et al., 2013). FGF21 protects kidney from damage by alleviating renal lipid accumulation and inhibiting inflammation, and fibrosis effects in diabetic nephropathy (Zhang et al., 2013). Through upregulating the expression of FGF21 and activating Akt2/GSK-3β/Fyn/Nrf2 antioxidants and the AMPK pathway, fenofibrate can exert a role in preventing diabetic nephropathy in the patients with type 1 diabetes (Cheng et al., 2020). In addition, activation of FGF21 pathway may correlate with the effect of SGLT2 inhibitors on protecting the renal function in type 2 diabetes and delaying progression of CKD (Packer, 2020).

## Fibroblast Growth Factor 21 and Chronic Kidney Disease

In 2009, Stein et al. (2009) reported the correlation between FGF21 and kidney disease for the first time, and found that the circulating FGF21 level of chronic hemodialysis patients increased by 15 times compared with the control group based on Caucasian population in a study from Germany. Subsequently, some studies have found an 8-fold increase of FGF21 in peritoneal dialysis patients compared to normal subjects according to a study from Korean (Han et al., 2010). In clinical practice, multiple studies have shown that the level of serum FGF21 is correlated with renal function of the patients with CKD (Crasto et al., 2012). Furthermore, the elevated plasma FGF21 level in the Chinese population significantly correlated with the state of CKD progression, and is independently linked to renal function and poor blood lipid levels (Lin et al., 2011).

The determination of FGF21 may help evaluate CKD and its complications, which is expected to become a relevant biomarker of CKD (Kuro-O, 2019a; Yamamoto et al., 2020). Serum FGF21 levels in CKD patients are positively associated with oxidative stress, and negatively associated with eGFR based on a cross-sectional study from Mexico (Ángel et al., 2021). The increase in FGF21 concentration in CKD patients may be related to the metabolism of lipids and carbohydrates, and FGF21 levels in CKD patients can be reduced through hemodialysis and transplantation from a Poland study (Marchelek-Myśliwiec et al., 2019). In peritoneal dialysis patients, FGF21 can be used as a hormone signal exerting a protective role in maintaining blood glucose homeostasis and preventing potential insulin resistance (González et al., 2016).

**FIGURE 2:**
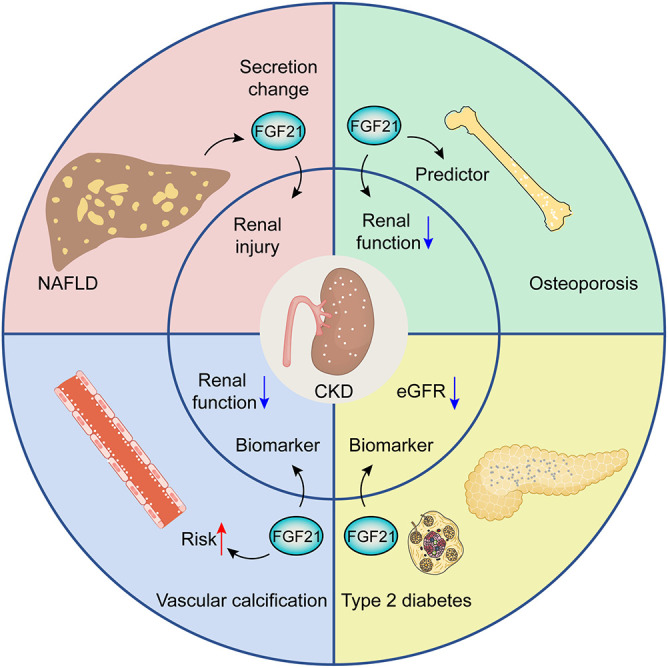
The potential roles of FGF21 in CKD patients with other pathological situations, including NAFLD, osteoporosis, diabetes, and vascular calcification.

There exert potential roles of FGF21 in CKD patients with other pathological situations (Figure 2). Growing evidence points to the potential interplay between non-alcoholic fatty liver disease (NAFLD) and CKD, the patients with NAFLD can result in renal injury by means of the alterations of FGF21 secretion (Musso et al., 2015). Similar findings have uncovered that FGF21 can serve as a biomarker for CKD progression and is associated with an increased risk of vascular calcification in CKD patients (Kuro-O, 2019a). FGF21 can be deemed as a sensitive predictor associated with osteoporosis in hemodialysis patients with worse renal function (Zhu et al., 2021). Serum FGF21 has been confirmed as a biomarker for predicting rapid progression of CKD patients with type 2 diabetes through eGFR decline (Looker et al., 2015).

Some studies have shown that acute kidney injury may accelerate the progression of CKD. Therefore, prevention of acute kidney injury is an important part of the treatment for CKD. In a mouse model of acute kidney injury induced by cisplatin, the application of recombinant FGF21 can remarkably downregulate the relevant protein levels of kidney injury (Li et al., 2018a; Chen et al., 2020). Additionally, another study demonstrates that the protective role of FGF21 in kidney injury can be induced by vascular calcification (Shi et al., 2018). Higher circulating FGF21 levels in patients with ESRD, but not with cardiovascular events, are associated with high mortality, which indicates that circulating FGF21 level can be used as a predictor for the prognosis of patients with CKD (Kohara et al., 2017). Although the metabolic disorder in CKD is usually thought to be the cause of the elevated FGF21, its precise mechanism has not been illustrated so far.

## Fibroblast Growth Factor 21 as a Potential Therapeutic Target

FGF21 has been clarified to have the effect on lowering blood sugar and lipids, so it is expected to be a potential candidate for development of CKD therapeutic. However, short half-life and poor stability are the bottleneck of clinical application of natural FGF21 protein. By constructing a stable mutant FGF21 (mFGF21) and then genetically fusing it with human albumin through a polypeptide to form HSA-mFGF21, whose half-life is 20 times higher than that of FGF21. It can enter the body to play a continuous inhibitory effect on blood glucose, which is expected to become a new biological therapy for metabolic disorders including diabetes (Watanabe et al., 2020). In addition, the FGF21 analog LY2405319 (LY) with the half-life improving exerts an inhibitory effect on blood sugar and lipids, which indicates that the FGF21 pathway may be an ideal candidate for the treatment of metabolic diseases (Adams et al., 2013). The new long-acting FGF21 analog PF-05231023 is a promising potential drug for the treatment of type 2 diabetes, obesity and obesity-related diseases (Weng et al., 2015; Thompson et al., 2016). The current research on targeting FGF21 therapy in CKD has certain limitations with a lack of corresponding clinical trials. At the same time, the current research results of some animal models may not be applicable to humans.

## Conclusion

**FIGURE 3:**
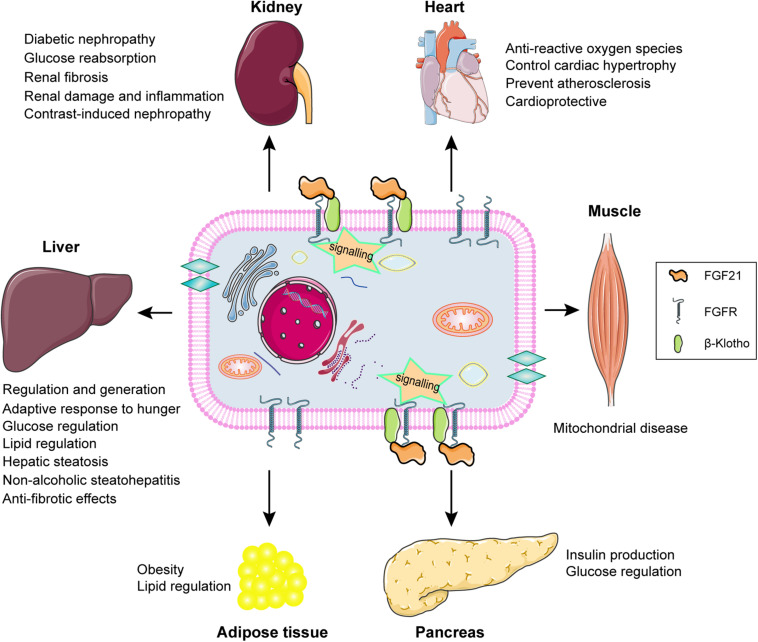
Schematic diagram of FGF21 signaling and the potential biological role of FGF21 in different types of tissue. FGF21 exerts potential biological roles in target organs, including liver, kidney, heart, muscle, pancreas, and adipose tissue, through binding to the βKlotho-FGFR complex achieving the signal transduction.

FGF21 signaling and the potential biological role of FGF21 in different types of tissue are summarized and displayed by the schematic diagram (Figure 3). FGF21 regulation performs the potential biological roles in different tissues based on FGFR-βklotho signaling transduction, including participating in the glucose and lipid regulation, the improvement of hepatic steatosis, and anti-fibrotic effects in liver, improving damage and fibrosis in kidney, preventing ROS, cardiac hypertrophy, and atherosclerosis in heart, predicting mitochondrial disease in muscles, regulating insulin production in pancreas, and lipid regulation in adipose tissue. The increase of FGF21 levels in CKD patients is influenced by a number of factors, and the pathophysiological significance and its positive or negative impact on patients have not been fully determined. It is possible to speculate that the level of FGF21 is adaptively increased in the early stages of CKD, which contributes to alleviate the metabolic disorders. As the severity of the continuous progress of CKD, the level of FGF21 is also rising correspondingly without more active role. Later the chronically elevated FGF21 may have adverse consequences for the patients with CKD. Consequently, FGF21 may become a potential target and blocking the effect of FGF21-βklotho endocrine axis may improve the curative effects on CKD patients. However, this needs more researches and clinical trials to further confirm.

## Author Contributions

XZ and NW contributed to the conception and design of the review article. XZ and YZ prepared the manuscript. NW revised the manuscript. All authors approved the final draft of the manuscript.

## Conflict of Interest

The authors declare that the research was conducted in the absence of any commercial or financial relationships that could be construed as a potential conflict of interest.

## Publisher’s Note

All claims expressed in this article are solely those of the authors and do not necessarily represent those of their affiliated organizations, or those of the publisher, the editors and the reviewers. Any product that may be evaluated in this article, or claim that may be made by its manufacturer, is not guaranteed or endorsed by the publisher.

## Abbreviations

CAG: coronary angiography
CIN: contrast-induced nephropathy
CKD: chronic kidney disease
eGFR: estimated glomerular filtration rate
ESRD: end-stage renal disease
FGF21: fibroblast growth factor 21
FGFR: fibroblast growth factor receptor
GLP-1: glucagon-like peptide-1
NAFLD: non-alcoholic fatty liver disease
NASH: non-alcoholic steatohepatitis
PPARα: peroxisome proliferator-activated receptor- α
ROS: reactive oxygen species
SGLT2: sodium glucose cotransporter 2
UGE: urinary glucose excretion.
